# Revisiting the Immunological Aspects of Temozolomide Considering the Genetic Landscape and the Immune Microenvironment Composition of Glioblastoma

**DOI:** 10.3389/fonc.2021.747690

**Published:** 2021-09-27

**Authors:** Natalia Di Ianni, Martina Maffezzini, Marica Eoli, Serena Pellegatta

**Affiliations:** ^1^ Unit of Immunotherapy of Brain Tumors, Fondazione IRCCS Istituto Neurologico Carlo Besta, Milan, Italy; ^2^ Unit of Molecular Neuro-Oncology, Fondazione IRCCS Istituto Neurologico Carlo Besta, Milan, Italy

**Keywords:** temozolomide, glioblastoma, immunotherapy, microenvironment, resistance

## Abstract

The microenvironment (ME) plays a critical role in causing glioblastoma (GBM) to be a moving and incurable target. The main features governing the interaction between cancer cells and the ME include dependency, promotion, and in rare cases, even competition. In the original Stupp protocol, the alkylating agent temozolomide (TMZ) is the first-line chemotherapy drug to treat GBM, and it is broadly used together or after radiotherapy. Some studies have described TMZ as an adjuvant to other therapeutic approaches including immunotherapy because of its ability to induce an immunogenic death of cancer cells. TMZ also exerts immunomodulatory effects on the tumor and immune ME. These findings support the coexistence of two circuits, i.e., one that subverts local immunosuppressive mechanisms and another that exerts a harmful influence on the peripheral immune response. A bias toward the latter can drive the failure of treatments based on the combination of chemotherapy and immunotherapy approaches. In this review, we will reanalyze how intrinsic and acquired resistance to TMZ impacts the immunomodulatory effects previously described by way of inducing a functional alteration of local immune cells and promoting immunosuppression and how different components of the immune ME, with particular attention to tumor-associated macrophages and microglia, can cause TMZ resistance to circumvent potential local immunogenic mechanisms.

## Introduction

Chemotherapy has historically been viewed as immunosuppressive until many studies have considered the immunomodulatory effects of conventional anticancer treatments (1). In 2012, Galluzzi described chemotherapy as “a secret ally for immunotherapy” based on the evidence that several standard chemotherapeutic compounds were able to enhance tumor-specific immune responses either by depleting immunosuppressive immune cells or by inducing the immunogenic death of tumor cells and modulating the tumor and immune microenvironment (ME) (2). In the last two decades, the limitations of chemotherapy and cancer immunotherapy as single therapeutic modalities have generated considerable interest in combining them to improve the outcome.

The treatment of glioblastoma (GBM), a devastating brain cancer with a dismal prognosis, is one of the most urgent unmet needs in oncology (3). However, temozolomide (TMZ), an alkylating chemotherapeutic agent, is still used as a standard treatment [Stupp regime (4)] and, in some clinical studies, in combination with immunotherapy as an adjuvant.

In evaluating the potential effect of TMZ on the tumor and immune ME, it is crucial to consider the heterogeneity of GBM and its resistance or sensitivity to TMZ and to evaluate the dynamics and genotype-dependent effects of the immune ME.

### Temozolomide as an Immunological Adjuvant: A Questionable Role

Some data support the idea that the role of TMZ in influencing host immune responses depends on its dosage. For example, in mice and humans, transient lymphodepletion due to the administration of increasing doses of TMZ enhanced a specific antitumor immune response induced by immunotherapy while simultaneously increasing the frequency of regulatory T cells (Tregs) (5). Furthermore, Karachi and colleagues described the effects of standard and metronomic TMZ combined with anti-programmed cell death protein 1 (PD1) therapy in modulating the frequency of peripheral immunosuppressive cells, including Tregs and MDSCs (6). An increase in the relative number of Tregs was observed in both treatments, and this effect was more pronounced after the standard dose of TMZ. Incidentally, the administration of the standard dose of TMZ was also implicated in the upregulation of programmed death-ligand 1 (PD-L1) expression on their surface (6) and, consequently, increased immunosuppressive activity (7).

Unlike the first evidence supporting the essential role of transient lymphodepletion in stimulating specific immune responses, recent studies have revealed that TMZ directly exerted a negative effect specifically on immune effector cells. In the preclinical model, the effect of anti-PD1 therapy was reversed by the systemic administration of 1,3-bis(2-chloroethyl)-1-nitrosourea (BCNU) and TMZ (8). The immunosuppressive effect of systemic chemotherapy, independent of the treatment dose, was observed as persistent peripheral lymphodepletion, poor infiltration of T cells within the ME, and a significant increase in Treg number compared to the controls. Conversely, anti-PD1 therapy combined with a local administration of chemotherapy, in the form of polymers loaded with the drug, demonstrated survival benefits due to the antitumor immune response within the ME (8). The advantage of the intratumoral administration of TMZ using a micro-osmotic pump in GL261 models was previously observed in combination with immunotherapy using granulocyte-macrophage colony-stimulating factor (GM-CSF), and this effect was based on the ability to trigger an immunogenic tumor cell death and, consequently, to favor an increased infiltration of effector cells into the tumor ME (9). Overall, in both studies, it was observed that the systemic administration of chemotherapy is immunosuppressive, whereas local chemotherapy enables the generation of a long-term antitumor T cell memory response induced by immunotherapy (8, 9).

The immunomodulatory effects of TMZ were also investigated in the GL261 model by systemically administering 10–13 times lower concentrations of the drug. The tumor ME was significantly skewed towards the pro-inflammatory direction in treated mice compared to the controls. The robust and persistent increase in local and peripheral activated NK cells was attributed to the resistance of NK cells to chemotherapy due to their unique ability to upregulate the multidrug resistance protein Abcc3 (ATP Binding Cassette Subfamily C Member 3) on their surface (10). In contrast, CD8+ T lymphocytes undergo apoptosis under TMZ treatment because they lack Abcc3 expression. Similar observations are reported in patients; GBM patients enrolled in the DENDR1 study (NCT04801147) were treated with dendritic cell (DC) immunotherapy combined with TMZ as an adjuvant, and data from this trial supported a negative effect of TMZ on peripheral CD8+ T lymphocytes and the generation of a memory status. A decline in CD8+ T cells was observed after the third vaccination when TMZ was concomitantly administered. The benefit on patient survival observed in a cohort of patients enrolled in DENDR1 was attributed to a significant and persistent activation of ABCC3-expressing NK cells (11), in agreement with observations from preclinical models (10). Furthermore, the expression and activity of ABCC3 in CD56^dim^ CD16^+^ NK cells under TMZ treatment were modulated by a specific single nucleotide polymorphism (-897DelC), which in turn correlated with a better clinical outcome (12). In another study, the expansion of antitumor immune responses was observed during DC vaccination in patients receiving dose-intensified TMZ as an adjuvant. However, the kinetics of absolute count and activation of CD8+ T cells showed a decline in vaccine response after the fourth vaccination. This effect was attributed to the potential negative impact of the continued concomitant administration of TMZ (13).

All these observations demonstrate that a considerable debate remains over the role of chemotherapy, such as TMZ, as an immunological adjuvant for immunotherapy treatments.

Considering that GBM is sustained by a unique, heterogeneous, and highly immunosuppressive ME that is also responsible for therapy resistance, in this review, we will focus our attention on 1) how chemotherapy can impact the composition of the immune ME in relation to the genetic and molecular landscape of GBM; and 2) how the immune ME constructed by brain resident and infiltrating immune cells, such as microglia and macrophages, is involved in the chemoresistance of GBM.

### Macrophages and Microglia, Crucial Protagonists in the Microenvironment

Malignant gliomas are typically characterized by an immunosuppressive ME in which macrophages and microglia are the most abundant components of innate immunity.

Circulating monocyte-derived macrophages (Mo) and tissue-resident microglia (Mg) are often described as GAMs (glioma-associated microglia/macrophages) that significantly contribute to tumor growth and invasion and immunosuppression due to their inadequate antigen-presenting capacity and suppression of T-cell proliferation. The percentage of GAMs present in GBM is positively correlated with tumor aggressiveness, as shown by their higher abundance in the mesenchymal (MES) molecular subtype (14–16).

GAMs can induce an MES-like state in cancer cells through ligand-receptor interactions, such as those between oncostatin-M and its receptor (OSM-OSMR), which have been well characterized by single-cell RNA sequencing (17). GAMs localize to different regions within GBM depending on their cell types: microglia preferentially reside at the tumor periphery; macrophages are recruited early within the tumor and reside in perivascular niches where direct interactions with tumor cells promote GBM growth and development (18, 19). The GAM composition is determined by a continuous spatial competition between Mo-GAMs and Mg-GAMs in a compensatory mechanism (20). The switch in GAM ontogeny is particularly evident in recurrent tumors after radiotherapy and chemotherapy with TMZ, which show the progressive loss of microglia and the increased infiltration and expansion of Mo-GAMs. A more inflammatory and hypoxic environment induced by therapy seems to facilitate Mo-GAM infiltration, probably because they adapt better to the environment than Mg-GAMs. *IDH* (Isocitrate DeHydrogenase) mutational status also impacts GAM subpopulation prevalence, resulting in a higher proportion of Mo-GAMs in more aggressive IDH wild-type gliomas (14, 16, 21).

### Temozolomide Resistance as a Critical Driver of an Immunosuppressive Microenvironment

Resistance to TMZ is one of the significant limitations in the treatment of GBM. GBM cells can be intrinsically resistant because they express DNA alkylating proteins and DNA repair enzymes. The most crucial factor involved in the resistance to TMZ is O-6-methylguanine-DNA methyltransferase (*MGMT*), whose expression is silenced by promoter methylation in 30%–50% of patients. Suppression of *MGMT* confers a greater sensitivity to TMZ and consequently improves survival (22). Lack of promoter methylation or high levels of MGMT proteins are associated with TMZ resistance (23, 24). Acquired resistance can also arise during treatment with TMZ, supporting that molecular events and cell signaling pathways can influence the response of GBM cells to chemotherapy. In this context, the immune ME can be considered critically involved in imparting resistance to therapy.

Many studies have described following differences in the immune contexture within the ME based on epigenetic modifications involved in the origin of chemoresistance.

#### Immune Contexture in Intrinsically Temozolomide-Resistant Glioblastoma

Recently, using a series of bioinformatics analyses, Zhao and colleagues found that unmethylated *MGMT* was associated with immune-related genes. Specifically, five genes, *GATA3* (GATA binding protein 3), *VDR* (Vitamin D Receptor), *TNFSF9* (TNF Superfamily Member 9), *TNFRSF9 (*TNF Receptor Superfamily Member 9*)*, and *LILRA5* (Leukocyte Immunoglobulin Like Receptor A5), were implicated in remodeling an immunosuppressive ME favoring M2 macrophage polarization. The expression of corresponding proteins was verified in GBM specimens by immunohistochemistry and the authors showed that they were significantly upregulated in GBM specimens with unmethylated *MGMT* compared with *MGMT*-methylated specimens (25). Chemoresistant glioma (GLTMZ) promotes M2-like polarization (CD11b+Gr1+CD68+CD206+) characterized by a significant increase in the release of immunosuppressive interleukin (IL)-10, which sustains tumor proliferation (26), CD206 expression, and arginase activity (proposed M2-like marker). Additionally, IL-10 can exert immunosuppressive effects by enhancing antioxidant enzymatic activity, thereby reducing the generation of anticancer reactive oxygen species (ROS) that mediate TMZ cytotoxicity and induction of immunogenic cell death. In a well-established crosstalk, M2-like macrophages educated by GLTMZ enhance *in vitro* and *in vivo* glioma proliferation (27). Macrophage and microglial reprogramming involves different mechanisms of alterations in cellular and molecular pathways, one of which is mediated by extracellular vesicles (EVs) released from GBM (28–30). EVs containing proteins, nucleic acids, and lipids contribute to creating a favorable tumor ME. Through cell-to-cell communication, tumor cells orchestrate immune responses mediated by the transfer of signaling molecules. EVs can induce an M2-like phenotype *in vitro* that promotes tumor recurrence and progression *in vivo* (30). TMZ can play a crucial role in promoting the release and modulation of EVs by GBM cells. Indeed, TMZ treatment of glioblastoma stem-like cells (GSCs) obtained from primary GBM resection specimens positively modulated the release of EVs and increased their number and amount of cargo. In particular, TMZ increased the levels of molecules related to cell adhesion and invasion and reduced patient survival (30, 31).

#### Immune Contexture in Acquired Temozolomide-Resistant Glioblastoma

In chemoresistance acquisition, changes in tumor metabolism play a key role, especially the switch from glycolysis to oxidative phosphorylation. TMZ resistance is conferred by the accumulation of superoxide dismutase 2 (SOD2), which regulates ROS levels and protects tumor cells against oxidative stress (32). The same metabolic shift is typical of M2 macrophages, whose glucose consumption is lower than that of M1-like macrophages, utilizes fatty acid (FA) oxidation, and is more dependent on mitochondrial oxidative respiration (33). In addition, TMZ-resistant tumors that rely on OXPHOS (mitochondrial oxidative phosphorylation system) promote the infiltration and enrichment of anti-inflammatory M2-like GAMs (CD45+CD11b+CD206+), particularly at the periphery of the tumor mass. These data support the hypothesis that TMZ-resistant GBM promotes a more immunosuppressive environment (34).

Phagocytosis of cancer cell by macrophages can restrict tumor growth and progression. Tumors adopt several immune escape mechanisms to avoid engulfment; one of these involves the Toll-like receptor 4 (TLR4). TLRs are key components of the innate immune system and, in the tumor ME, are expressed by both tumor and immune cells (31). TLR4 expression is representative of M1 polarization, and its signaling is required to stimulate the phagocytic functions of macrophages and induce an inflammatory response. TLR4 was found to be particularly downregulated in chemoresistant GBM and macrophages cocultured with GBM cells. Chemoresistant GBM cells promote the suppression of TLR4 expression in tumor-infiltrating macrophages that release cytokines such as IL-6 and IL-10 and support a tumor-promoting ME. This mechanism could represent an immune escape strategy carried out by tumor cells (35).

### Immune Microenvironment as a Crucial Player in Resistance to Temozolomide

In GBM, the heterogeneity of the immune contexture within the ME can impact the effects of TMZ.

GAMs not only drive the formation of immunosuppressive ME but also induce GBM stemness and enhance *in vitro* and *in vivo* tumorigenicity. Furthermore, human microglia can promote TMZ resistance by the augmented expression of inflammatory IL-11, which activates the STAT3 (signal transducer and activator of transcription 3)-MYC pathway in tumor cells (36). STAT3 activation is implicated in therapy resistance independent of *MGMT* methylation and in shaping the tumor ME by promoting M2 polarization in a signaling loop of immunosuppression and immune escape. PI3K (PhosphatidylInositol 3-Kinase)-γ inhibition reduces IL-11 accumulation in the tumor ME, which mimics the TMZ response of the so-called “exceptional responders” whose tumors show a reduced trafficking and infiltration of GAMs (36). The connection between the chemotherapy response and microglia is similarly evident from the capacity of TMZ-resistant GBM cells to promote M2 polarization by increased levels of lncRNA (long noncoding RNA) SNHG15 (small nucleolar RNA host gene 15). In addition, M2 microglial cells expressing CD163 and CD206 are recruited by IL-10, IL-4, IL-13, and CCL2 and promote GBM proliferation by secreting transforming growth factor (TGF)-β and IL-6 (37).

The major histocompatibility complex (MHC) II chaperone CD74 is a cell-surface receptor for the cytokine macrophage migration inhibitory factor (MIF). Some data suggest that CD74 has a pro-tumorigenic role. In particular, gliomas may escape the pro-inflammatory M1 conversion of macrophages/microglia *via* CD74 activation by MIF, a mechanism that contributes to shape the contexture of GBM (38–40). MIF is predictive of poor outcomes and early tumor recurrence in GBM, and its secretion by self-renewing cancer stem cells (CSCs) promotes immunosuppression mediated by MDSCs. Ha et al. demonstrated that MIF and CD74 expression are implicated in mediating resistance against TMZ in GBM. Indeed, the specific MIF inhibitor Ibudilast improves the survival in *in vitro* and *in vivo* models by sensitizing tumors to TMZ treatment (41). CD74 was also identified as a potential modulator of TMZ responsiveness, and its shRNA-mediated knockdown, in *MGMT* methylated glioma cells, significantly increased sensitivity to chemotherapy (42, 43). Kitange et al. confirmed a higher level of CD74 mRNA expression in cells derived from TMZ-resistant xenografts; CD74 modulates the TMZ response, probably through the mitogen-activated protein kinase (MAPK) and protein kinase B or Akt (PKB/Akt) signaling pathways, whose activation promotes cell proliferation and survival (43).

The tight association between GBM cells and GAMs in chemoresistance was confirmed by Kazantseva et al. in a study on the TP53 isoform Δ133p53β, which has pro-tumorigenic functions such as the induction of tumor growth and migration, protection from apoptosis, promotion of angiogenesis, and reduction of chemotherapy responsiveness. In addition, this isoform reduces sensitivity to TMZ treatment and promotes cell survival by reducing oxidative stress in an immunosuppressive ME characterized by increasing the recruitment of CD163 and colony stimulating factor 1-receptor (CSF1-R) positive macrophages mediated by CCL2 (44).

## Conclusions

GBM is an urgent unmet medical need. Genetic driver mutations of tumor cells, biological and molecular heterogeneity of the tumor, and immune ME can impact the response to chemotherapy administered as a standard or adjuvant therapy.

**Figure 1:**
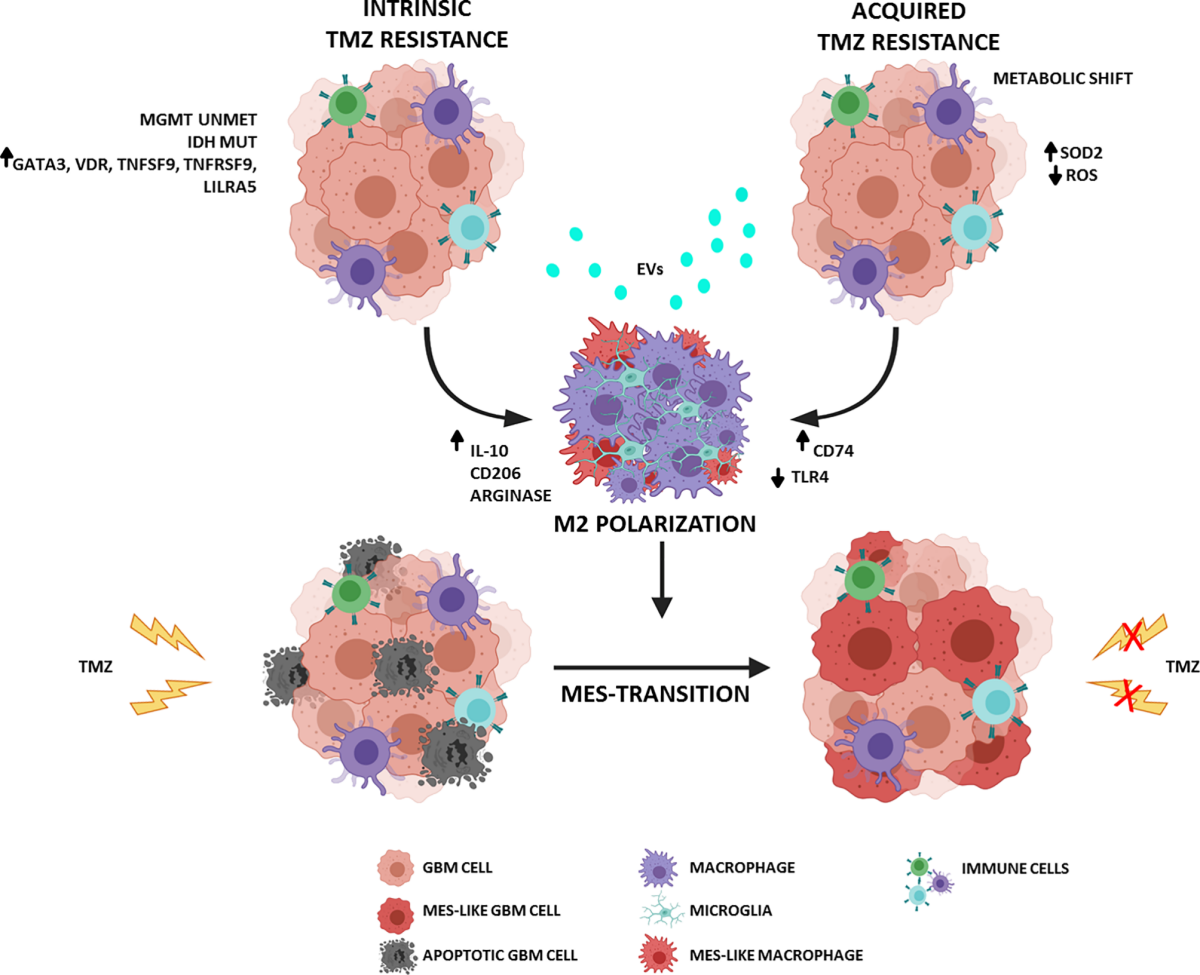
Schematic representation of the interplay between GBM and ME in TMZ resistance. (Figure adapted from images created with BioRender.com).

Despite the promising role of TMZ in treating newly diagnosed GBM, it has not proven to be effective against recurrent GBM. The dynamic interplay between GBM cells and immune ME influences many aspects of tumor evolution: survival, proliferation, recurrence, and therapeutic response. Therapy sensitivity is not governed only by a few molecular pathways but is modulated by intrinsic and acquired factors that involve and/or depend on ME. In a significantly immunosuppressive context, macrophages can play a crucial role in TMZ resistance and GBM relapse (**Figure 1**). Consequently, the most recent efforts are focused on characterizing the ME with particular attention to infiltrating innate immune cells, including macrophages and microglia. Understanding such complexity of biological systems requires an extensive analysis of “single” cell subpopulations.

In recent months, several studies have explored the immune cells within the ME of GBM by using single-cell RNA sequencing technologies, with special attention to the reciprocal communication between macrophages and tumor cells. Macrophages can induce the transition of GBM to mesenchymal (MES)-like tumors, which are considered as the more aggressive molecular subtype of GBM (17). In this context, macrophages are sculpted by MES-GBM cells, which can enhance their mesenchymal program. The adaptability of macrophages imposes another critical limitation in efficient antitumor rewiring of metabolic signals. In IDH1-mutant gliomas, R-2-hydroxyglutarate (R-2-HG) significantly decreases the antigen-presenting signature in macrophages and induces an immunosuppressive phenotype (45). On the other hand, single-cell analyses revealed that a higher proportion of microglia than macrophages was beneficial and correlated with a better prognosis in GBM patients independent of the *MGMT* status (46).

The wealth of information on microglia and macrophages can be exploited to overcome the challenges of combinatorial targeted therapies. Looking forward, standard therapeutic protocols, including TMZ, will be harmonized and combined with innovative (immuno)therapeutic approaches that take dynamic heterogeneity of tumor cells and macrophage/microglia into consideration. In addition, immune modulation of ME by reprogramming macrophages/microglia can reverse chemoresistance and improve TMZ efficacy.

## Author Contributions

Writing—original draft preparation: NI, MM, and SP. Review and editing: ME and SP. All authors contributed to the article and approved the submitted version.

## Conflict of Interest

The authors declare that the research was conducted in the absence of any commercial or financial relationships that could be construed as a potential conflict of interest.

## Publisher’s Note

All claims expressed in this article are solely those of the authors and do not necessarily represent those of their affiliated organizations, or those of the publisher, the editors and the reviewers. Any product that may be evaluated in this article, or claim that may be made by its manufacturer, is not guaranteed or endorsed by the publisher.
